# The Influence of Using Steel Tapes and Composite Materials on Reinforcing Hot-Rolled Steel Profiles

**DOI:** 10.3390/ma17133086

**Published:** 2024-06-23

**Authors:** Ilona Szewczak, Patryk Rozylo

**Affiliations:** 1 Faculty of Civil Engineering, Lublin University of Technology, 40 Nadbystrzycka Str., 20-618 Lublin, Poland; 2 Faculty of Mechanical Engineering, Lublin University of Technology, Nadbystrzycka 36, 20-618 Lublin, Poland; p.rozylo@pollub.pl

**Keywords:** hot-rolled steel profiles, CFRP tapes, reinforcement method, adhesive connection

## Abstract

Steel structure designers frequently encounter the need to reinforce hot-rolled compressed steel elements. This is particularly common in the case of compressed truss bars in steel truss girders. Typically, reinforcement is designed using bars or flat bars welded to the compressed element. However, welding technology is not always feasible in existing and operational steel halls due to fire safety concerns. To address this challenge, researchers investigated alternative reinforcement methods using bonded steel and CFRPs (carbon fiber-reinforced polymers/plastics) tapes. Laboratory tests and numerical analyses were conducted. Eleven 1.5 m long specimens made of 50 × 50 × 4 angle iron from S235 steel were subjected to axial compression testing. The test samples included three unreinforced samples, three samples reinforced with steel tape bonded using SikaDur-30 adhesive, and five samples reinforced with CFRP tape (SikaDur-30 adhesive was used for bonding in three cases, and 3M VHB GPH-160GF tape in two cases). The research conducted indicates that reinforcement using bonded steel tapes is the most effective method for limiting vertical displacements and deformations, as well as increasing the load-bearing capacity of the tested angles by 28.6% compared to the reference elements. Considering the high cost of composite tapes, this is valuable information from an economic analysis perspective. The absence of steel tape delamination suggests that the bonding technique can be successfully employed in this reinforcement method and can replace welding, for example in facilities where there is a high fire hazard.

## 1. Introduction

The development of steel structures necessitates the exploration of innovative reinforcement techniques for steel structural components, driven by factors such as increased external loads or design flaws. In existing structures, reinforcing steel structures using welding or mechanical fasteners may not always be feasible. Adhesive steel or composite tapes offer a promising approach for rapid and non-invasive reinforcement of steel structural elements.

In the available scientific publications, there are works dedicated to the topic of strengthening axially compressed steel profiles. This is in response to frequent inquiries from the industry about the possibility of reinforcing existing steel structures, such as compressed rods in steel truss girders. The publications discuss studies of axially compressed steel profiles reinforced by welding additional elements. The authors of [1] presented tests of compressed steel angles strengthened directly by welding and analyzed the influence of weld spacing on the strength of the sample. Weld spacing had essentially no effect on the experimental strength of the test specimens when the weld spacing was less than or equal to 305 mm on center. The works [2,3] describe the strengthening of steel elements of lattice towers through the use of steel angles and mechanical connectors. The aim of the research was to investigate the influence of interconnections on the efficiency of load transfer between the core and the strengthening elements. The experimental results also confirm the effectiveness of the retrofitting method. Axial loads can be effectively transferred between the original steel members and the reinforcing members via a bolted connection system. In turn, one work [4] presents an analytical solution for strengthening a pre-loaded steel angle L120 × 120 × 12 and 2.5 m long using a second angle with an identical cross-section and connected with battens. A review of the literature revealed few works describing the reinforcement of compressed steel elements by gluing tapes or composite mats. They mainly concerned the reinforcement of steel profiles with a closed cross-section (reinforcement of thin-walled steel columns with a square cross-section axially compressed using CFRP tapes [5] and mats [6] or channel sections [7,8]). At the same time, no publications were found on the reinforcement of axially compressed steel angles reinforced by gluing steel or composite tapes, although these profiles are very often used in truss roof trusses, which is extensively described in the work [9] (in which the failure loads of trusses made of angle sections, determined using various design methods, were compared with the test results).

In this study, research on the reinforcement of axially compressed hot-rolled steel angle profiles with carbon fiber-reinforced polymer (CFRP) tapes and steel tapes is described. The use of adhesive bonding techniques for reinforcing existing steel structures can be a helpful solution in active facilities where welding processes are not possible (and reinforcement using mechanical connectors is impossible or too time-consuming). Additionally, comparing the effects of reinforcement using composite tapes and steel tapes can enable preliminary conclusions regarding the economic rationale for using composite tapes.

## 2. Preparation for the Tests

**Figure 1:**
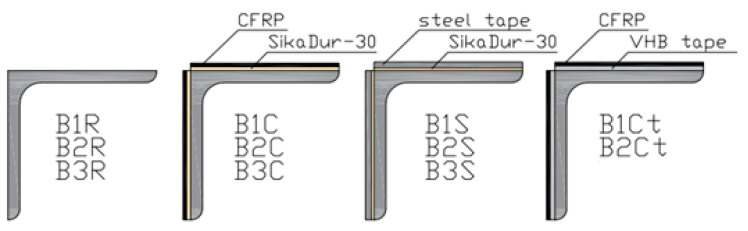
The laboratory test sample symbols.

Eleven samples with a length of 1.5 m made from a 50 × 50 × 4 angle of S235 steel were subjected to axial compression testing. The first three samples were reference samples, named B1R, B2R, and B3R. All other samples were reinforced by gluing steel tapes or CFRP tapes with a length of 1.3 m to the external surfaces of the angle arms, so that both ends of the samples remained unreinforced over a length of 10 cm. Three samples (B1C, B2C, B3C) were reinforced by gluing CFRP Sika CarboDur S512 composite tapes with a cross-section dimension of 50 × 1.2 mm to the external surfaces of the angle arms using SikaDur-30 adhesive, with an adhesive layer thickness of 1.3 mm. Three more samples (B1S, B2S, B3S) were reinforced using strips of steel sheet with a cross-section dimension of 50 × 2 mm made of S235 steel. SikaDur-30 adhesive was used for the connection, and the adhesive layer thickness was 0.9 mm. The last two samples (B1Ct, B2Ct) were reinforced with CFRP Sika CarboDur S512 composite tapes, which were glued to the surfaces of the angles using 3M VHB GPH-160GF tape with a width of 50 mm and a thickness of 1.6 mm. Before proceeding with the reinforcement, all samples were cleaned and roughened using sandpaper P120 and degreased with extraction gasoline. The nomenclature of the beams and the method of reinforcement are shown in Figure 1, while the basic parameters of the materials used are presented in Table 1.

**Figure 2:**
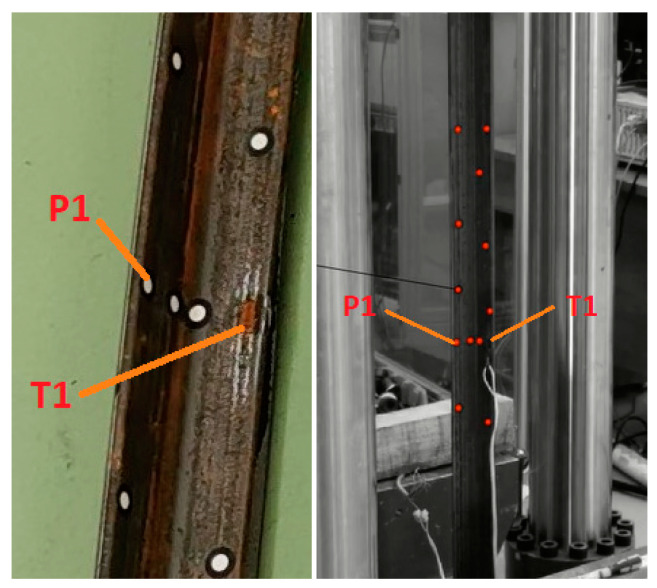
Placement of the electrical resistance strain gauge (T1) and the displacement measurement point (P1) in the middle of the beam’s height.

Laboratory tests were initiated after a minimum of 7 days following the reinforcement to obtain the full strength parameters of the SikaDur-30 adhesive. Before commencing the tests, all samples were checked for preliminary geometric imperfections. No curvature was found along the length of the sample. The next step was to apply a strain gauge on the inner surface of the angle bracket arm, in the middle of the sample span and at a distance of 5 mm from the edge of the cross-section, to measure strains. Additionally, measurement points were applied on the inner surface of the angle bracket, where displacement measurements were taken. Figure 2 shows the location of the measurement point P1, where the displacement results are described in the work, and the electrical resistance strain gauge T1.

## 3. The Laboratory Tests

**Figure 3:**
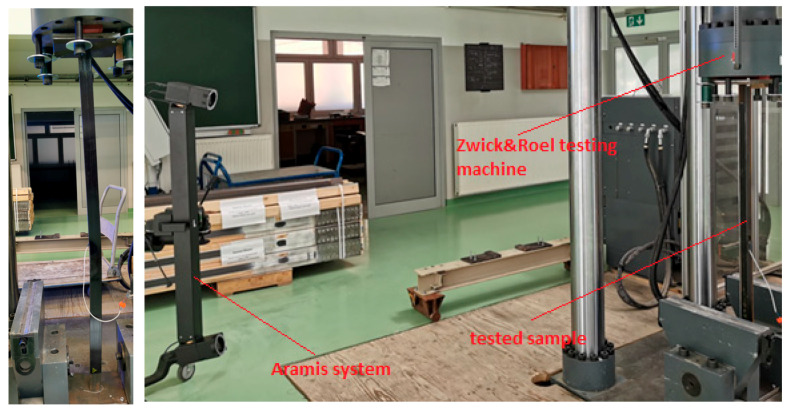
The photos of the test stand.

Tests in the axial compression scheme were conducted on a Zwick&Roel (ZwickRoell GmbH & Co. KG, Ulm, Germany) strength-testing machine. The load was generated by the movement of the press piston at a rate of 2 mm/min, recording the load increase every 0.01 s. Samples were placed in specially prepared holders. In a 10 mm thick steel plate, holes corresponding to the cross-section of the tested angle brackets were cut out. Samples were placed in the cut-out hole, which caused rigid mounting, and were subjected to loading in the strength testing machine. During the test, strains were measured using TENMEX TFs-10 120 Ω ± 0.2% electrical resistance strain gauges and displacement measurements at control points using the Aramis system. The use of the Aramis system allowed for the measurement of sample displacements in 3D. Photos of the test setup are shown in Figure 3.

## 4. Laboratory Test Results

**Figure 4:**
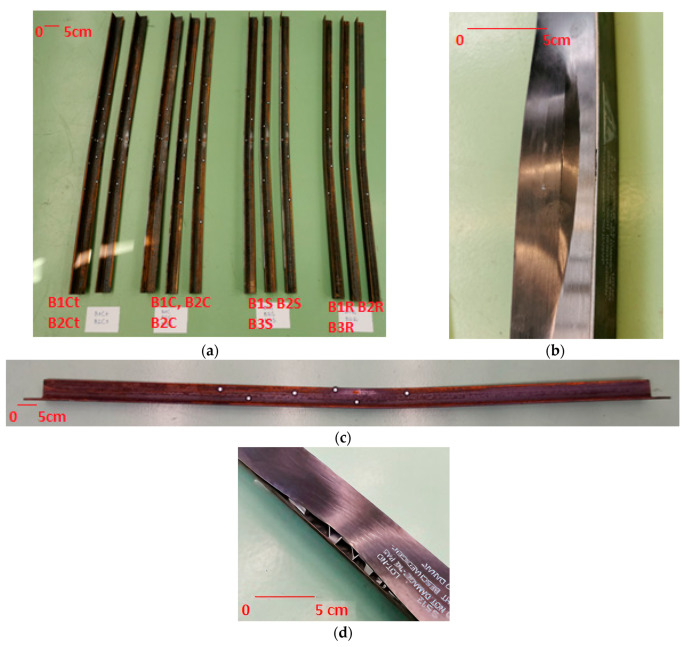
Method of sample damage: (**a**) all samples after testing, (**b**) cracking of the CFRP tape in sample B1C, (**c**) method of beam deformation after testing (the white points are stickers for measuring displacements using the Aramis system), (**d**) detachment of the CFRP tape mounted with VHB tape.

The samples were subjected to a load until failure. During the test, the samples experienced buckling. Despite significant displacement values in the case of samples reinforced with steel tape and CFRP tape glued with SikaDur-30 adhesive, no detachment of the reinforcing tape occurred in any sample (only in sample B1C did the CFRP tape crack but only after achieving maximum load). In samples reinforced with CFRP tape using 3M VHB GPH-160GF tape, detachment of the tape was observed, but only after achieving the maximum load value. The image of the sample damage is shown in Figure 4.

**Figure 5:**
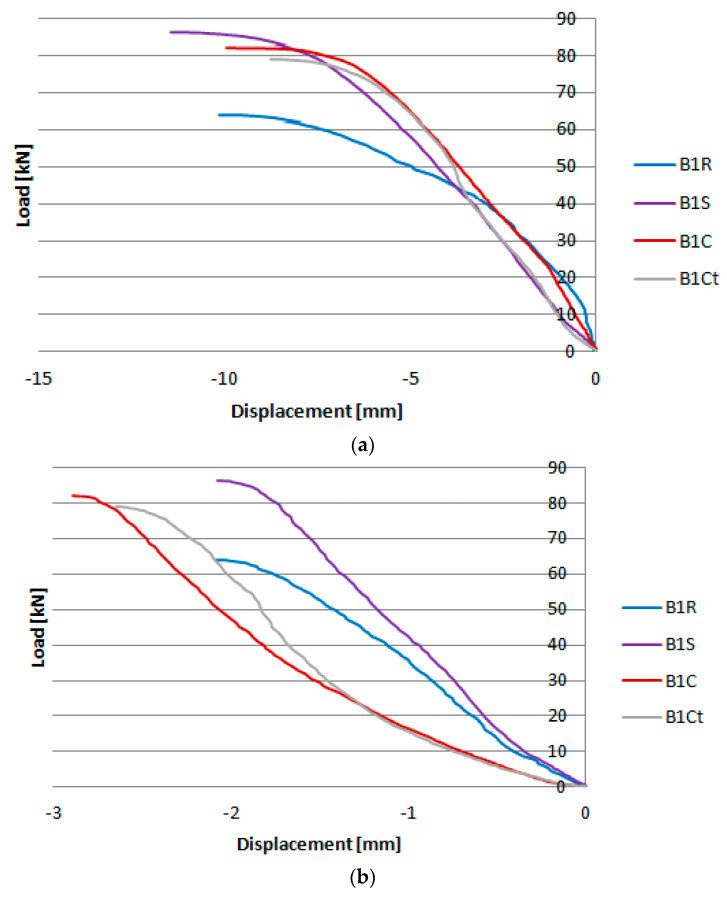
Results of measuring the displacements of selected beams in the (**a**) horizontal and (**b**) vertical direction.

**Figure 6:**
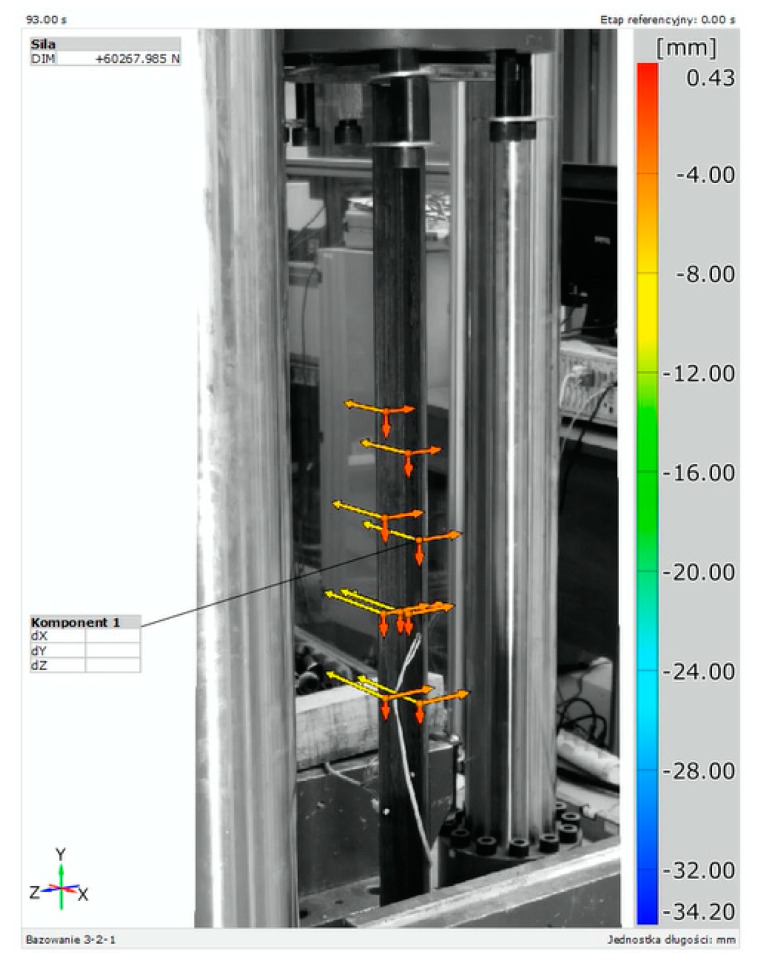
An example image of a displacement measurement obtained from the GOM Correlate program.

During the test, displacement measurements in horizontal and vertical direction at point P1 and strains using an electrical resistance strain gauge T1 were taken in all samples. The location of the measurement points on the cross-section in the middle of the span of the samples is shown in Figure 2. Due to the large number of samples, presenting all the results on the charts would be illegible. Therefore, the charts (Figure 5) show the horizontal (Figure 5a) and vertical (Figure 5b) displacement results read at point P1 for one sample from each group, while the detailed test results are compiled in Table 2. Figure 6 shows an exemplary image of displacements obtained from the GOM Correlate program, based on measurements made at points in the middle of the beam span using the Aramis system.

In order to formulate conclusions from the research, it was decided to present the results of displacements and strains for groups of samples with a given method of reinforcement, at a load level of 60.3 kN. This is due to the fact that after exceeding this load value, some of the electrical resistance strain gauges were destroyed, and the obtained results were unreliable. The obtained results are presented in Table 3. 

Analyzing the data presented in Table 3, it can be seen that the use of each method of reinforcement contributes to a significant reduction in horizontal displacements and strains. In the case of limiting vertical displacements and strains, the most effective turned out to be the use of reinforcement with steel tapes bonded with SikaDur-30 adhesive. In this case, it is worth emphasizing that the much stiffer steel tapes did not detach from the surface of the samples. In the case of horizontal displacements, the most advantageous turned out to be the use of CFRP tapes bonded with SikaDur-30 adhesive. The application of CFRP tapes bonded onto VHB tapes also contributed to a significant reduction in horizontal displacements, which confirms the very good strength parameters of VHB tapes and may encourage further laboratory research on the reinforcement of steel elements using them.

## 5. Finite Element Method Simulations

Numerical models were developed with the best possible representation of actual structures using Abaqus^®^ software (Abaqus 2023, Dassault Systemes Simulia Corporation, Velizy Villacoublay, France). Numerical simulations were conducted using the finite element method (FEM). Numerical calculations were performed in two stages: the first stage was the solution of the so-called linear eigenproblem, while the second stage was a non-linear analysis of the stability of the construction. In the course of the first stage of the investigation, only the buckling forms of the structure in axial compression were determined, which were then implemented in the second stage of the non-linear calculations. The first stage was limited only to solving the linear problem of structural stability according to the relationship [10,11,12]:

(1) $$\left(K_{0}^{NM}+\lambda_{i}K_{\Delta }^{NM}\right)\nu_{i}^{M}=0$$

where $K_{0}^{NM}$ is the stiffness matrix (equivalent to the baseline condition—includes the preloads effects *P^N^*), $K_{\Delta }^{NM}$ is the differential initial stress and load stiffness (resulting from the incremental loading pattern *Q^N^*), $\lambda_{i}$ represents the eigenvalues, $\nu_{i}^{M}$ constitutes the buckling form—eigenvectors, *M* and *N* are the degrees of freedom of the model, and *I* is the buckling mode. Additionally, the buckling load is defined using the following equation: $P^{N}+\lambda_{i}Q^{N}$.

The second, and also the main stage of the analyses considered the implementation of the initial buckling form obtained into the non-linear structural stability calculations. In the second stage, the behavior of the structure was analyzed in the full range of loading—from the initial phase of loading the structure, up to the loss of load capacity.

Simulations of non-linear stability analysis were performed using the incremental-iterative Newton–Raphson numerical technique [13,14]. The above-mentioned method allowed for the determination of the post-equilibrium paths, enabling a better assessment of the stability state:

(2) $$F^{N}\left(u^{M}\right)=0$$

where *F^N^* denotes the load component conjugate to the *N*th variable and *u^M^* denotes the value of the *M*th variable.

The FEM simulation generally uses the Newton–Raphson technique for solving the non-linear equilibrium equations. For the aforementioned method, after an iteration *i*, an approximation *u_i_^M^* to the solution has been obtained. Regarding the above, let *c*_*i*+1_*^M^* be the difference between this solution as well as the exact solution to the discrete equilibrium equation:

(3) $$F^{N}\left(u_{i}^{M}+c_{i+1}^{M}\right)=0$$

If the left side of the above equation is expanded, it will take the following form:

(4) $$F^{N}\left(u_{i}^{M}\right)+\frac{\partial F^{N}}{\partial u^{P}}\left(u_{i}^{M}\right)c_{i+1}^{P}+\frac{\partial^{2}F^{N}}{\partial u^{P}\partial u^{Q}}\left(u_{i}^{M}\right)c_{i+1}^{P}c_{i+1}^{Q}+\ldots =0$$

When *u_i_^M^* comes as a very close approximation to the solution, the magnitude of any *c_i+_*_1_*^M^* will be negligible, so all of the above expressions except the first two can be neglected, resulting in a linear system of equations:

(5) $$K_{i}^{NP}c_{i+1}^{P}=-F_{i}^{N},\;\;\;\;K_{i}^{NP}=\frac{\partial F^{N}}{\partial u^{P}}\left(u_{i}^{M}\right),\;\;\;\;F_{i}^{N}=F^{N}\left(u_{i}^{M}\right)$$

The next approximation to the solution is then as follows:

(6) $$u_{i+1}^{M}=u_{i}^{M}+c_{i+1}^{M}$$

In this way, the iteration continues. The above-presented relationships present the algorithm of the method used for the non-linear analysis.

Consequently, the numerical models developed were able to represent the actual behavior of structures in axial compression [13,14]. The numerical models were developed using available programs such as Abaqus^®^ software (Abaqus 2023, Dassault Systemes Simulia Corporation, Velizy Villacoublay, France). A numerical model of a steel beam named BR was initially prepared. It was a beam made as a solid model using C3D8R-type finite elements (linear shape function elements, 8 nodes, having 3 translational degrees of freedom at each node, with reduced integration). In addition, the elements constituting the beam mountings were also modelled, with special grooves into which the ends of the beam profile were placed. The beam mountings were modelled using non-deformable finite elements of type R3D4 (four-node elements, with 6 degrees of freedom). For the second type of beam named BS, the model of the beam itself and the mountings were identical. In addition, this model used longitudinal reinforcing steel strips, which in turn were modelled using C3D8R-type finite elements—the same as for the base beam model. In the case of the third beam model, named BC, the beam and mountings were the same as the previous cases, and additional reinforcing composite strips were modelled using S4R-type finite elements (shell elements with a linear shape function, having 4 nodes in each element, with reduced integration). In the case of the BR-type numerical model, the mesh of the numerical model consisted of 27,688 finite elements and 35,300 computational nodes. For the other two models of the BS and BC types, the numerical values of the mesh finite elements were 43,288 and 32,888, respectively, while the computational nodes were 58,268 and 41,042.

As for the mesh density used and the size of the finite elements for the discretization process, the chosen mesh sizes allowed the results to be as close as possible to the experimental results. The global mesh density for the compressed structure was 5 mm, while additional care was taken to have a minimum of 3 elements by thickness. This approach made it possible to obtain results as close as possible to the experimental studies. Another research paper analyzed the effect of mesh density on the accuracy of the calculation results, and slight differences in the density magnitude do not significantly affect the results—which is further presented in the scientific publication [15].

Within the framework of the calculations carried out for the base material of the beam, i.e., S235 steel, an elastic–plastic material model was used based on the material data presented in the description of the research subject in this publication. The steel reinforcements were made of identical material. For CFRP composite strips, an orthotropic material model was used according to the information described in the research subject. For the numerical simulation, no adhesive connection was analyzed—all joint relationships, between the beam and the steel or composite reinforcements, were modelled using a permanent non-separable connection of the TIE type, available in Abaqus^®^ software. This approach made it possible to speed up the calculation procedure and simulate the issue of loss of stability and load carrying capacity over the full load range, without losing the convergence of calculations. Contact interactions—in the normal and tangential directions with a friction coefficient of 0.2—were used between the ends of the steel beam end sections and the mountings.

**Figure 7:**
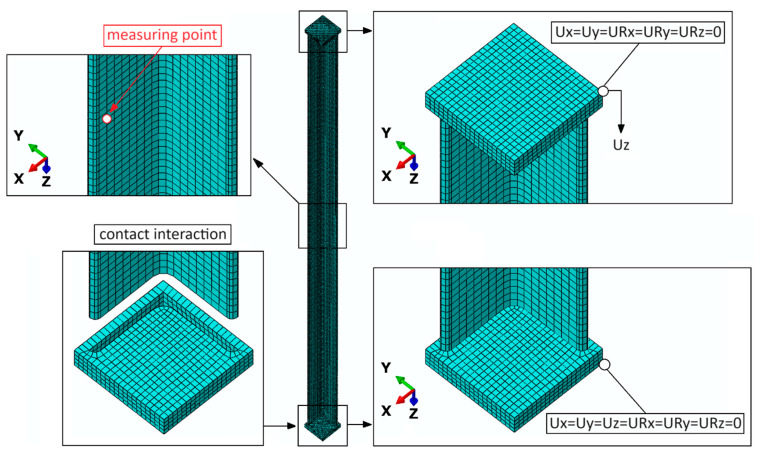
The numerical model with boundary conditions.

The boundary conditions of the numerical model made it possible to simulate the phenomenon of axial compression of the structure. Regardless of the type of test subject used (BR, BS or BC), the loss of stability and load-carrying capacity of the structure were simulated, with an estimation of the value of the limit force, after which the structure loses the ability to continue to carry the compressive load. The boundary conditions and discrete model of the example structure are presented in the figure below (Figure 7). During the numerical simulations, nodal displacements (in all X, Y and Z directions) were measured at the point shown in the figure below.

## 6. Comparison of Laboratory Test Results and Numerical Analyses

**Figure 8:**
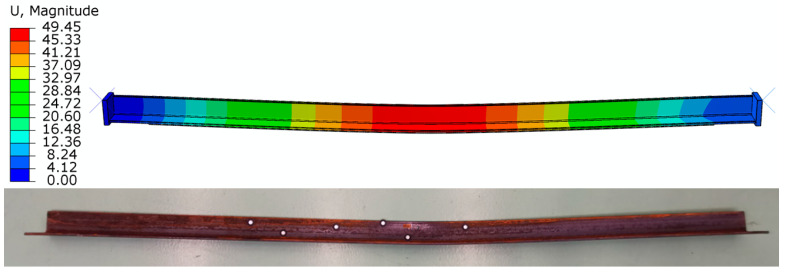
Mode of deformation of samples during laboratory tests and in numerical analysis.

Comparing the developed numerical models with samples tested in the laboratory, it was found that both for the model of the sample without reinforcement and for the samples reinforced with CFRP tapes and steel tapes, a satisfactory convergence of results for displacements and maximum force was achieved. Figure 8 shows the deformation of the sample after laboratory testing and during numerical analysis in the Abaqus program.

**Figure 9:**
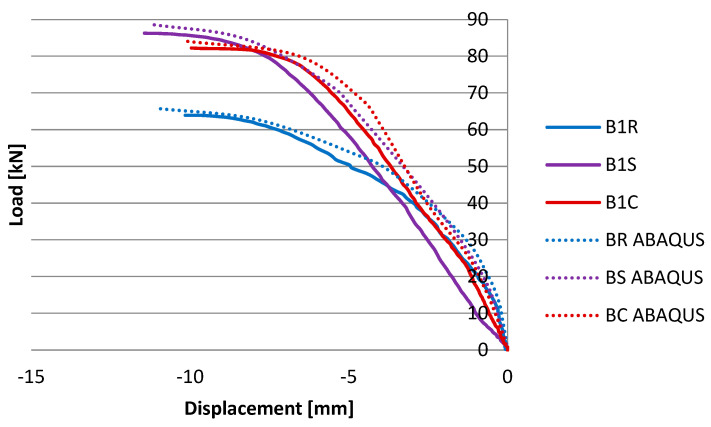
Load–displacement relationship chart in the horizontal direction for individual numerical models (with results of EXP tests).

The results of horizontal displacements depending on the load value for individual numerical models are presented in the chart (Figure 9). However, to present a clear comparison of the laboratory research results and numerical analyses, Table 4 was developed.

The largest discrepancy between the obtained maximum force value is 5.5% (BC, BC ABAQUS). In the case of the displacement measurement, the maximum discrepancy between the obtained results was 9.9% (P1 horizontal, BS, BS ABAQUS), while in other cases it oscillated around 6%. The numerical model obtained lower values of horizontal displacements and higher values of the maximum force (except for BR). The numerical model has been validated as optimally as possible, and the differences may result from, for example, dimensional imperfections of the tested beams. As can be seen in Table 2, there was a dispersion of results between individual samples in a given group.

The displacement results of individual numerical models developed during the numerical analyses indicate that reinforcement with steel tapes allows for limiting vertical displacements and an increase in the maximum destructive force value by nearly 26% compared to the reference model. To limit displacements in the horizontal direction, both in laboratory studies and the numerical analyses, the most effective appears to be the reinforcement with CFRP tapes (according to the numerical analyses, there was a reduction in displacements in this direction by over 44%, and according to laboratory tests by almost 43%).

## 7. Final Conclusions

The laboratory and numerical studies conducted herein confirm the high effectiveness of reinforcements of axial compressed hot-rolled steel angle using bonded CFRP tapes and steel tapes. In none of the tested samples did the reinforcing tapes detach before reaching the maximum failure force, despite quite large displacement values, which indicates that the gluing technique can effectively replace, for example, welding technology in this case.

The results obtained during laboratory tests indicate the following:

- The use of reinforcement with bonded steel tapes using SikaDur-30 adhesive allows for a reduction in vertical displacements by 19.3%, horizontal displacements by 32%, and strains by 32.6% (under a load of 60.3 kN) and an increase in the maximum value of the destructive force by 28.6% compared to reference samples;
- Similarly, the use of reinforcement with bonded CFRP tapes using SikaDur-30 adhesive allows for a reduction in horizontal displacements by 42.7% and strains by 21.3% (under a load of 60.3 kN) and an increase in the maximum value of the destructive force by 19.7% compared to reference samples; however, it does not affect the reduction in vertical displacements;
- The use of reinforcement with bonded CFRP tapes on VHB tape allows for a reduction in horizontal displacements by 18% and strains by 5.5% (under a load of 60.3 kN) and an increase in the maximum value of the destructive force by 12.5% compared to reference samples; however, it does not affect the reduction in vertical displacements.
- The results obtained during the numerical analyses are close to the results of the laboratory tests (in the vast majority of measurements, the difference does not exceed 6%).

The studies conducted herein indicate that the execution of reinforcement in the form of bonded steel tapes allows us to limit vertical displacements and strains to the greatest extent and to increase the load capacity of the tested compressed steel angle by 28.6% compared to reference elements. Considering the high cost of composite tapes, this is very valuable information from an economic analysis perspective (the time to complete the reinforcement using glued CFRP tapes and steel tapes is the same, while in Poland the cost of purchasing 1 m of CFRP tape is 17 times higher than the purchase of 1 m of steel tape). The lack of delamination of steel tapes indicates that the gluing technique can be successfully used in this reinforcement method and replace the welding technique, for example, in facilities where there is a high fire hazard.

The obtained efficiency of reinforcement for samples reinforced with CFRP tape bonded on VHB tapes was not as significant (an increase in load capacity by 12.5%); however, due to the very fast and simple execution and the lack of damage to the VHB tape before achieving the maximum load value, it encourages further research into such a reinforcement technique. Also from an economic point of view, when significant strengthening of the steel element is not required, it is worth taking a look at this method of strengthening because the cost of purchasing VHB tape and tape glue is similar, while the labor cost is much lower and does not require the use of additional tools for mixing and applying glue.

## Figures and Tables

**Table 1:** The basic parameters of the materials.

CFRP Sika CarboDur S512 tape	
Poisson’s ratio	ν = 0.308
Young’s modulus	E = 165 GPa
Steel tape	
Poisson’s ratio	ν = 0.3
Young’s modulus	E = 210 GPa
Yield strength of steel	235 MPa
Tensile strength of steel	420 MPa
SikaDur-30 adhesive	
Minimum compressive strength after 7 days	75 MPa
Compressive modulus	9600 MPa
Minimum tensile strength after 7 days	26 MPa
Minimum shear strength	16 MPa
Minimum peel strength after 7 days	21 MPa
Shrinkage	0.04%
VHB GPH-160GF	
Foam density	710 kg/m³
90° Peel adhesion to stainless steel after 72 h	34 N/cm
Dynamic shear	375 N/6.54 cm²
Normal tensile after 72 h	470 N/6.54 cm²

**Table 2:** Results of measuring displacements and strains of the tested samples.

	Load [N]	P1 Vertical [mm]	P1 Horizontal [mm]	$T1\;[\times 10^{-6}$]	Sample Group Symbol	Average Value of the Maximum Load [N]
B1R	60,300	−1.78	−7.43	−	BR	66,476
	63,920	−2.08	−10.15	−		
B2R	60,300	−1.61	−6.28	−1544		
	70,601	−2.10	−10.18	−1839		
B3R	60,300	−2.19	−7.98	−1539		
	64,908	−2.54	−11.52	−1827		
B1S	60,300	−1.37	−5.20	−1026	BS	85,504
	86,278	−2.08	−11.44	−2071		
B2S	60,300	−1.74	−5.20	−1165		
	83,853	−2.39	−10.61	−1767		
B3S	60,300	−1.40	−4.37	−922		
	86,379	−2.05	−10.39	−1730		
B1C	60,300	−2.28	−4.55	−1170	BC	79,579
	82,233	−2.89	−9.96	−2423		
B2C	60,300	−1.79	−5.06	−1253		
	76,184	−2.46	−12.18	−		
B3C	60,300	−1.53	−2.83	−		
	80,320	−2.20	−9.38	−		
B1Ct	60,300	−2.02	−4.60	−1387	BCT	74,765
	78,993	−2.64	−8.75	−3624		
B2Ct	60,300	−3.12	−7.26	−1527		
	70,537	−3.61	−10.61	−1925		

**Table 3:** Results of displacement and strain measurements of the tested samples under a load of 60.3 kN.

Sample Group Symbol	P1 Vertical [mm]	P1 Horizontal [mm]	T1 $\left[\times 10^{-6}\right]$
BR	−1.86	−7.23	−1541
BS	−1.50	−4.92	−1038
BC	−1.87	−4.14	−1212
BCt	−2.57	−5.93	−1457

**Table 4:** Comparison of numerical and laboratory research results.

Results of Laboratory Tests				Results of Numerical Analyses			
Sample Group Symbol	P1 Vertical [mm]	P1 Horizontal [mm]	Average Maximum Load [N]	Sample Group Symbol	P1 Vertical [mm]	P1 Horizontal [mm]	Maximum load [N]
BR	−1.86	−7.23	66,476	BR ABAQUS	−1.89	−7.07	65,773
BS	−1.50	−4.92	85,503	BS ABAQUS	−1.4	−4.43	88,793
BC	−1.87	−4.14	79,579	BC ABAQUS	−2.05	−3.93	84,189
	average displacement values with a load of 60.3 kN				average displacement values with a load of 60.3 kN		

## Data Availability

Data are contained within the article.
